# Intermittent fasting reduces interictal epileptiform discharges and hippocampal reactive astrogliosis during electrical kindling epileptogenesis

**DOI:** 10.1007/s11011-025-01607-9

**Published:** 2025-04-15

**Authors:** Josué Denichi Sánchez-Hernández, Joaquín Manjarrez-Marmolejo, Octavio Fabián Mercado-Gómez, Angélica Vega-García, Javier Franco-Pérez, Virginia Selene Arriaga-Ávila, Sandra Orozco-Suárez, Rosalinda Guevara-Guzmán

**Affiliations:** 1 Neurodegenerative Disease Experimental Laboratory, National Institute of Neurology and Neurosurgery “Manuel Velasco Suárez”, Zip Code #14269, Tlalpan, CDMX México; 2 Department of Physiology, Faculty of Medicine, National Autonomous University of Mexico, Zip Code #70250, Coyoacán, CDMX México; 3 Neurological Diseases Medical Research Unit, Specialty Hospital, National Medical Center Siglo XXI, Mexican Social Security Institute, IMSS, “Dr. Bernardo Sepúlveda”, Zip Code #6720, Cuauhtémoc, CDMX México; 4 Laboratory for Cerebral Vascular Pathology, National Institute of Neurology and Neurosurgery “Manuel Velasco Suárez”, Zip Code #14269, Tlalpan, CDMX México

**Keywords:** Astrogliosis, Epileptiform discharges, Fasting blood glucose, Hippocampus, Intermittent fasting, Kindling

## Abstract

It has been proposed that interictal epileptiform discharges observed in epilepsy-related structures, such as the hippocampus contributes to epileptogenesis. Intermittent fasting (IF) produces anticonvulsant effects protecting against experimentally induced seizures. It has been suggested that protective effects could be associated with astrocytic metabolic changes. However, it is unknown whether IF modifies the epileptogenic process. Therefore, this work aimed to analyze the impact of IF on interictal epileptiform discharges in the rat hippocampus and their possible correlation with astrocytic activation. Male Wistar rats were divided into two groups: one group had free access to food, while the other group was subjected to IF (food was provided for 2 h a day). Both groups underwent a hippocampal electrical kindling protocol with 36 stimulations. Our results showed that IF inhibited the incidence of generalized seizures induced by kindling and significantly reduced the total spectral power of interictal epileptiform discharges in the hippocampus. Furthermore, IF prevented the rise in fasting blood glucose and reduced seizure-induced astrogliosis by preventing the GFAP expression and the morphological complexity of astrocytes in the hippocampal CA3 region. These results support the notion that IF modifies epileptogenesis by modulating hippocampal hyperexcitability during the interictal stage, which could be associated with reductions in glucose metabolism and astrogliosis.

**Graphical Abstract:**

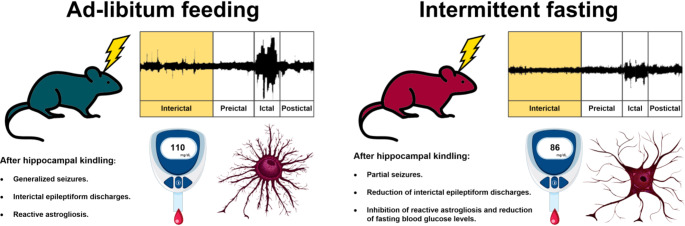

**Supplementary Information:**

The online version contains supplementary material available at 10.1007/s11011-025-01607-9.

## Introduction

Epilepsy is a neurological disorder characterized by seizures with a high incidence in the world population (Beghi 2020). Seizures in epileptic patients can be divided into four stages characterized by the appearance of abnormal patterns of brain activity. The preictal stage corresponds to the period preceding the onset of an epileptic seizure, whereas seizure per se is denoted as the ictal stage. The postictal stage refers to the recovery period occurring immediately after the active part of the seizure, and the interictal stage comprises the period between seizures (Fisher et al. 2014; Yang et al. 2024). Distinguishing and analyzing the four stages is essential to determine the cyclic nature of seizures and could contribute to the diagnosis, treatment, and management of this disease (Staba and Worrell 2014). The interictal epileptiform discharges, or interictal spikes, are generated by the synchronous activity of neuronal populations. These discharges are a distinctive feature of the epileptic brain because they arise between seizures and are not associated with behaviors or motor manifestations (Vossel et al. 2013; Lee et al. 2023). Therefore, it has been proposed that interictal epileptiform discharges, recorded in structures such as the hippocampus, can be associated with the development of epilepsy (epileptogenesis), the generation of seizures (ictogenesis), and functional alterations in memory consolidation (de Curtis and Avanzini 2001; Staley and Dudek 2006; Lambert et al. 2020). Thus, detecting and analyzing interictal activity can provide crucial information on neuronal excitability and seizure predisposition (Fisher et al. 2014; Scott et al. 2021; Holmes 2022).

On the other hand, the potential of intermittent fasting (IF) as a dietary treatment for drug-resistant epilepsy is a promising avenue of research. A few reports have shown that IF induces anticonvulsant effects similar to those described for the ketogenic diet (Landgrave-Gómez et al. 2016; Magdy et al. 2022), leading to its proposal as a potential treatment (Yuen and Sander 2014). However, there is a lack of studies describing whether IF can inhibit or delay epileptogenesis or report the mechanisms underlying the inhibition of seizures once the epileptogenic process is well established. A hypothesis related to this phenomenon proposes that the cerebral effects of IF could be modulated by astrocytes, which play an essential role in regulating energy metabolism in the brain (Silva et al. 2005). These cells transport and deliver nutrients to neurons, control cerebral blood flow in an energy activity-dependent manner, and store glycogen as the major source of glucose in the brain (Beard et al. 2022). By contrast, astrocytes in pathological conditions exhibit reactive astrogliosis, a change that affects their complexity and is characterized by increasing glial fibrillary acidic protein (GFAP) expression (Zhou et al. 2019).

To date, few preclinical reports have focused on recognizing and analyzing interictal electrical activity in models of epileptogenesis (Bortel et al. 2010). Therefore, we used a hippocampal kindling electrical model to generate seizures in rats and to investigate the effects of IF on epileptiform discharges during the interictal stage and their possible correlation with astrocytic activation.

## Materials and methods

### Electrodes implantation

**Fig. 1:**
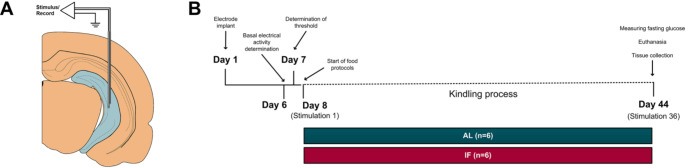
**A**) Scheme showing the recording and stimulating electrodes in the hippocampus. **B**) Experimental design. AL: Ad Libitum (*n* = 6), IF: Intermittent fasting (*n* = 6)

Male Wistar rats weighing between 280 and 300 g were used. The experimental protocol was approved by Ethical and CICUAL (Ethical CICUAL number 005-CIC- 2020) committees following the official Mexican standard (NOM- 062 ZOO- 1999) for the care and use of laboratory animals. Animals were anesthetized intraperitoneally with a mixture of ketamine/xylazine (90/10 mg/kg). To record local field potentials (LFPs), a bipolar stainless-steel microelectrode (diameter = 100 μm, impedance less than 4 mΩ) for stimulation and recording was implanted deep in the CA3 region of the right hippocampus (AP: − 5.4 mm, L: 5.0 mm, V: − 6.0 mm) (Fig. 1 A). The electrode was soldered to a micro-USB connector and fixed to the skull with dental cement. The animals were allowed free access to water and food for seven days after implantation. The antibiotic enrofloxacin (5 mg/kg i.p.) was administered during the first three postoperative days. At the end of the experimental period, the implantation site of the electrodes was confirmed using a cresyl violet stained coronal brain section (the technique described by Paxinos and Watson 2007), and animals that were implanted outside the correct coordinates were discarded (Supp. Figure 1 A).

### Intermittent fasting and kindling model

Two groups were established; one was allowed to free access to food and water (ad libitum, AL) and the other was subjected to IF (*n* = 6 per group). The IF consisted of allowing rats to feed freely for only two hours daily (from 12 to 2 pm) for 36 days (Rivera-Zavala et al. 2011) (Fig. 1B). Additionally, a third group was established as a sham group, in which the animals were implanted with electrodes but not subjected to electrical stimulation or metabolic treatment.

Hippocampal kindling stimulation was used as a model of epileptogenesis according to a protocol modified from that of Becker and colleagues (1997). This electrical model allowed us to evoke seizures and clearly differentiate each seizure stage, thus analyzing the effects of IF on LFPs during the interictal stage. First, the current threshold was determined by connecting the animals to an amplifier (BE Light, EBNeuro, Italy) coupled to a Grass S88 electrostimulator and then applying a 50 Hz monophasic square-wave electrical stimulus for 1 s, starting at 20 µA and increasing by 10 µA. In this way, the electric current necessary to evoke an afterdischarge with a minimum duration of 5 s was determined. This threshold determination showed us that there were no significant differences between groups because the stimulation current was 83.3 ± 8.0 µA in the AL group and 86.7 ± 8.4 µA in the IF group.

The kindling stimulation and dietary protocols were started for the next day, as illustrated in Fig. 1B. Rats were stimulated for 36 days with their respective threshold current, with a stimulus applied once a day (6:00–9:00 am). In this regard, previous work in our laboratory established that under the stimulation conditions described, a maximum of 36 stimuli are required to induce a fully kindled state in healthy animals, without IF treatment (data not shown). Therefore, the IF group was stimulated daily after a 16-hour of fasting. The LFPs from the hippocampal CA3 region and seizures evoked by kindling were recorded and stored in a computer for later evaluation (EEG-video). EEG video was monitored 20 min before stimulation and 20 min after stimulation. The inclusion criteria used to ensure optimal signal quality for subsequent analysis were as follows: (a) The voltage of the electrical activity must be twice the baseline. Baseline activity was defined as the electrical signal recorded before the day of the kindling threshold determination, i.e., in animals that had not yet undergone any experimental manipulation (Fig. 1B). (b) The signal must be free of artifacts. (c) The animals must be awake since slow-wave sleep (SWS) can present seizure brain activity patterns on the EEG, which could lead to misinterpretation. Blind observer, who was unaware of the treatments applied to each rat, analyzed, and classified the seizures according to the Racine scale (Sutula and Kotloski 2017). Score 0, no seizure; score I, facial myoclonus (blinking of one eye) with head movements accompanied by wet dog shaking; score II, chewing; score III, myoclonus of one forelimb; score IV, myoclonus of both forelimbs supported by the hindlimbs; score V, loss of balance followed by generalized seizure. Animals were fully kindled if they exhibited five score IV seizures or three score V seizures on consecutive days.

### Hippocampal LFPs analysis

**Fig. 2:**
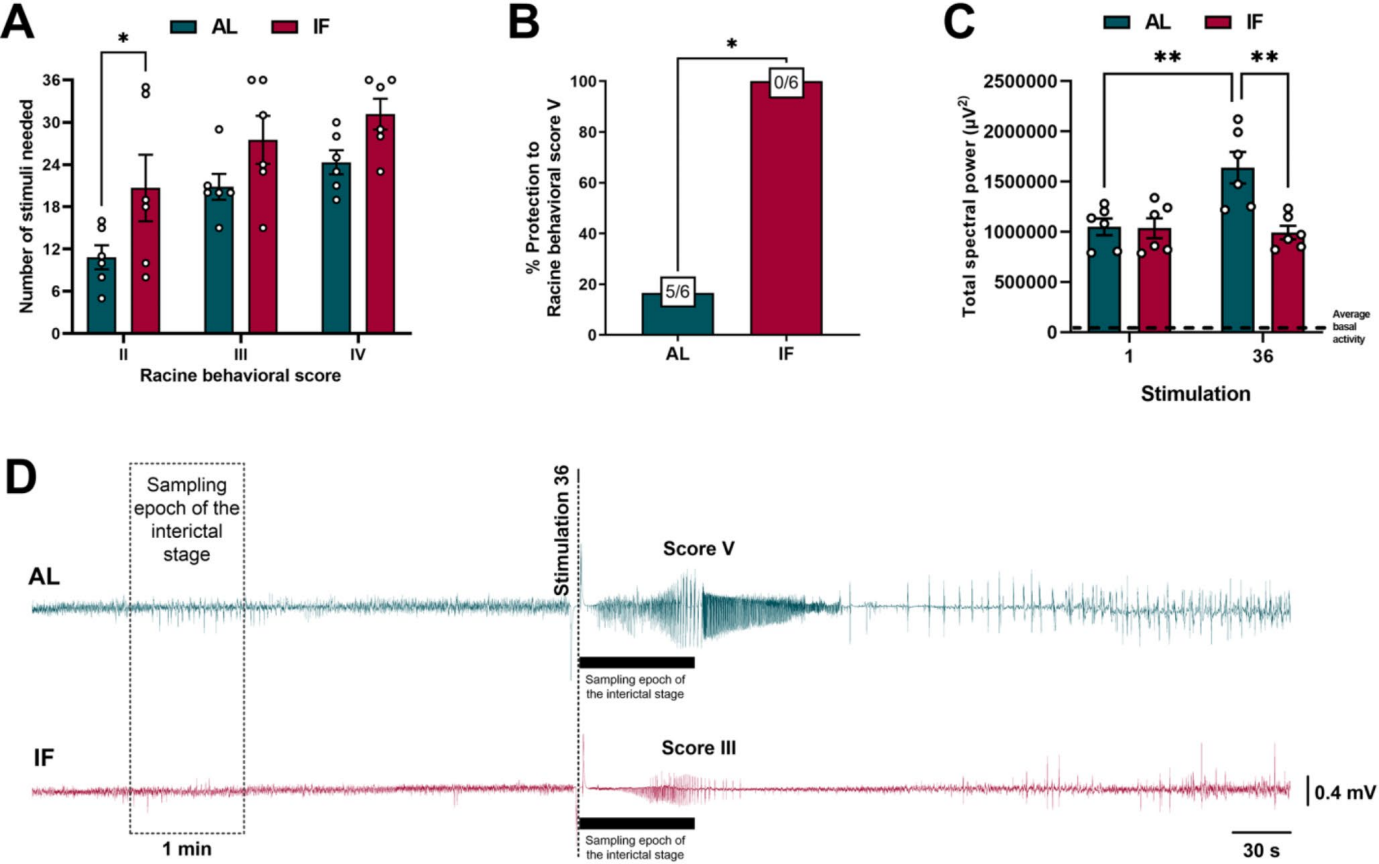
**A**) Stimuli needed to achieve each behavioral score on the Racine scale. Data are expressed as median ± SEM. Two-way repeated measures ANOVA was used followed by Tukey’s post hoc test, *p < 0.05. **B**) Percent protection to Racine score V; note that no IF-treated animal reached the criterion of being fully kindling. Fisher’s exact probability test, *p < 0.05. **C**) Total spectral power of the hippocampal LFPs in the ictal state on the first day of stimulation and on the last day of stimulation. Data are expressed as median ± SEM. Two-way repeated measures ANOVA was used followed by Tukey’s post hoc test, **p < 0.01. D) Representative recordings of LFPs in AL (blue) and IF (red) animals at stimulus 36 to induce epileptogenesis. Sampling epochs of the interictal stage and the after-discharge produced by the stimulation are remarked. Note the presence of interictal epileptiform discharges before the application of electrical stimulation, which were not related to seizure behavior

On the final stimulation day (stimulus 36), rats were connected to the amplifier and hippocampal LFPs were recorded. During the interictal phase (5 min before the kindling stimulation) and ictal phase (immediately after the kindling stimulus), 1-minute epochs were extracted from the recordings for the following analysis (Fig. 2D). The LFPs were sampled at a frequency of 256 Hz, and the signal was filtered from 0.3 Hz to 30 Hz using Galileo NT software version 3.0 (Italy). For the interictal signal, the interictal spikes present at the previously selected sampling times were analyzed. The duration of the interictal discharge was determined from the beginning of each event until the return to baseline (Bortel et al. 2010). It is characterized by a high-amplitude paroxysmal depolarizing shift (> 50 µV) lasting up to 100 ms (Lai et al. 2023). The amplitude and number of spikes per minute were measured, and their morphology was determined.

The interictal and ictal electrical signals (1-minute sampling epochs) were subjected to analysis using the Fourier Transform (FT) to decompose the signal into its frequency components and quantify its spectral power. The analysis was conducted using MATLAB software (MathWorks Inc., USA) in conjunction with the EEGLAB interactive toolbox (University of California, San Diego, USA). This software performs the analysis using the Discrete Fourier Transform (DFT), which was calculated using the Fast Fourier Transform (FFT) (Delorme and Makeig 2004):

$$\:X\left(f\right)=\sum\:_{n=0}^{N-1}{x\left(n\right)e}^{-j2\pi\:fn/N}$$

Where:

X(f) is the frequency spectrum of the EEG signal.

(𝑛) is the signal in the time domain.

*N* is the total number of samples in the window.

𝑒^−j2πfn/N^ is the Fourier kernel, which decomposes the signal into its frequency components.

The result is a frequency and amplitude function that describes the spectral distribution of the EEG. Additionally, the signal frequency (Hz) and the relative spectral power of the different bands were determined: delta (0–4 Hz), theta (4–8 Hz), alpha (8–12 Hz), and beta (12–30 Hz) (Sánchez-Hernández et al. 2022).

### Physical and liver weight measurement and plasma albumin assessment

To investigate whether IF had any impact on the animals’ body weight, we recorded the weight at the beginning and conclusion of the kindling stimulation protocol (corresponding to stimuli 1 and 36, respectively). To verify that IF did not cause malnutrition in animals, we evaluated the concentrations of plasma albumin, a nutritional marker. Briefly, the animals were euthanized with an overdose of anesthesia, and blood samples were collected intracardially using a 10-ml syringe. Then, 5 ml of blood was transferred to Vacutainer^®^ blood collection tubes with 1 ml of EDTA solution (110 mM), and the blood samples were centrifuged for 10 min at 3,000 rpm at room temperature. Using plastic pipettes, plasma was transferred to a clean plastic screw-cap tube and stored at − 80º C until use. Albumin levels were quantified from plasma sample dilution using proper kits (Avivia OKIA00131, USA) following the manufacturer’s instructions. Finally, the livers from the AL and IF groups were perfused with saline, dissected, and weighed to examine whether dietary restriction could affect the size of the organ.

### Blood glucose and β-hydroxybutyrate levels measurements

Glucose supplementation before seizure generation has been found to reduce the anticonvulsant effects of the ketogenic diet in mice (Mantis et al. 2014). Furthermore, in treatments based on restrictive diets (i.e. calorie or time-restricted feeding), β-hydroxybutyrate has been identified as one of the key metabolites contributing to the anticonvulsant effects associated with these dietary regimens (Landgrave-Gómez et al. 2016). Therefore, we decided to evaluate blood glucose and β-hydroxybutyrate levels on the last day of stimulation (under fasting conditions) to determine whether there was a relationship between glucose, β-hydroxybutyrate levels, and the predictable antiepileptogenic effects of IF. For this purpose, we analyzed blood glucose and β-hydroxybutyrate using the Precision Xtra monitoring system (Abbott Laboratories). The measurement was automatically obtained by this device using a blood drop sample from the rat’s tail 20 min before the last electrical stimulation (16 h of fasting).

### Immunofluorescence for astrocytes and morphological analysis

At the end of the experimental period, animals were euthanized with pentobarbital (150 mg/kg) (Pisa, Guadalajara, JC, Mexico) and perfused intracardially with phosphate-buffered saline (PBS) solution (pH 7.4) followed by 4% paraformaldehyde (Sigma-Aldrich, St. Louis, MO, USA) in PBS. Brains were removed and post-fixed in 4% paraformaldehyde for 24 h and then placed in increasing concentrations of sucrose (10, 20, 30%) for three consecutive days at 4 °C. Coronal Sect. (10 μm) were obtained from the entire ventral hippocampus using a cryostat (Leica, Germany). The obtained tissue slices were stored at − 20 °C in an antifreeze cryoprotectant solution (10% ethylene glycol, 40% glycerol in PBS, pH 7.4) (Sigma-Aldrich, St. Louis, MO, USA) for further processing. Brain tissues were processed for immunofluorescence to observe the morphological changes of astrocytes by analyzing the expression of GFAP after kindling stimulation. Tissues were mounted on slides treated with poly-L-lysine and washed with PBS and 0.2% Triton X- 100 (J.T. Baker, USA). Brain tissue sections were blocked with normal goat serum (1:200, Vector Laboratories) in PBS for 15 min and incubated with a primary rabbit polyclonal antibody to GFAP (1:200, Z0334, Agilent Dako) in PBS at 4 °C for 48 h. Then, they were rinsed with PBS for 5 min, incubated with Alexa 594-conjugated anti-rabbit (1:300, A11062, Thermo Fisher Scientific, USA) in PBS for 3 h, and washed with PBS for 5 min. Finally, the sections were covered with Fluoromount G mounting medium (17984 - 25, Electron Microscopy Sciences).

Images were acquired using a Nikon T1 Eclipse inverted confocal microscope and NIS Elements v.4.50 software. The fluorophore was excited sequentially with the integrated laser line 594 nm. The corresponding fluorescence was read in the 500–550 nm range using filters provided by the manufacturer. Photomicrographs were taken at 20 × (2 sections per rat). The Skeleton (2D/3D) plug-in of ImageJ software (NHI) was utilized to quantify morphological parameters (number of branches and junctions) of astrocytic cells (50–60 individual cells per group).

### Statistical analysis

All data were tested for normality using the Shapiro-Wilk test. Fisher’s exact probability test was used to compare protection against generalized seizures (Racine score 5). The number of stimuli required to achieve each behavioral score on the Racine scale, spectral power, number of interictal spikes, interictal spike duration and amplitude, LFP frequency, and body weight were analyzed using a two-way repeated measures ANOVA followed by Tukey’s post hoc test. In addition, the relative power of frequency bands was analyzed using the Kruskal-Wallis test. Student’s t-test was used to evaluate the liver weight, albumin levels, fasting blood glucose, and β-hydroxybutyrate levels. The relationships between blood glucose levels, hippocampal activity in the interictal phase, and the Racine behavioral index were determined by simple linear regression followed by Pearson and Spearman tests, respectively. Data on fluorescence levels of GFAP and morphological data of astrocytes were analyzed using the Mann-Whitney U test. Significant differences were considered in all tests when *p* < 0.05 was obtained. Statistical tests were performed using Prism 8.0 (GraphPad Software).

## Results

### The IF protects against generalized seizures induced by hippocampal kindling

We found that the AL group required fewer stimuli to reach each Racine behavioral score than the IF group. Specifically, the AL group needed 10.8 ± 1.7 stimuli to reach a score of 2, while the IF group needed 20.7 ± 4.7 stimuli. Furthermore, to consider that the animals had reached the kindling state (five scores out of four in a row without reaching a score of five on the Racine scale), the AL group required 24.3 ± 1.7 stimuli, compared to the IF group which needed 31.2 ± 2.1 stimuli. However, this data was not statistically significant (Fig. 2 A). It is noteworthy that none of the rats in the IF group exhibited generalized tonic-clonic seizures (Racine score 5) following daily electrical stimulation of the hippocampus for 36 days (Fig. 2B). During the ictal stage evoked by hippocampal stimulation, we were able to detect highly synchronous activity, or interictal epileptiform discharges, in AL rats. This was characterized by LFPs with higher voltage and duration compared to those observed in the hippocampus of rats subjected to IF (Fig. 2 C and D).

### IF reduces hippocampal interictal epileptiform discharges during epileptogenesis

**Fig. 3:**
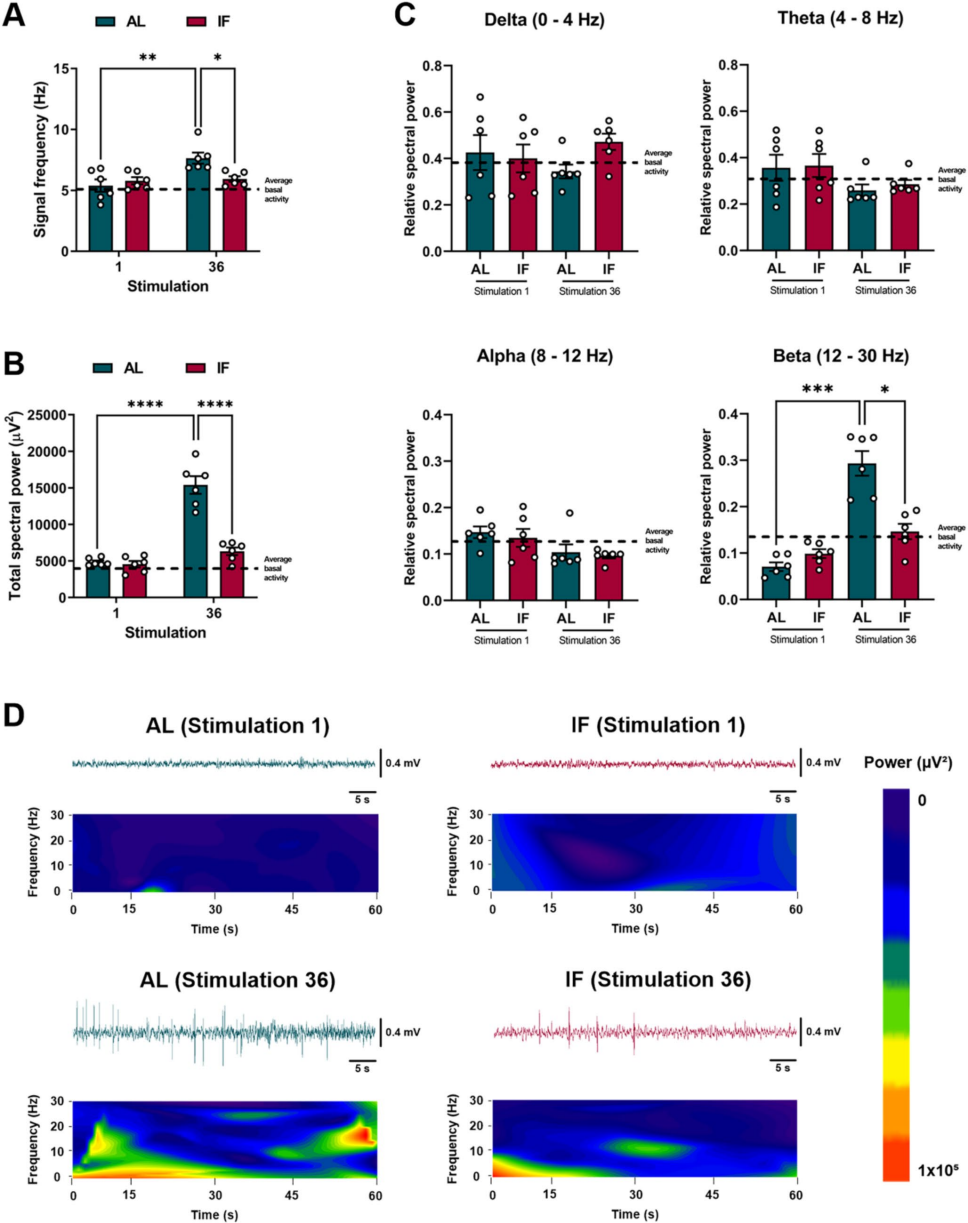
**A**) Frequency of the hippocampal LFPs in the interictal stage (before kindling stimulation) on the first and last stimulation day. **B**) Total spectral power of the hippocampal LFPs in the interictal stage on the first and last stimulation day. **C**) Relative spectral power of delta (0–4 Hz), theta (4–8 Hz), alpha (8–12 Hz), and beta (12–30 Hz) bands in the interictal stage on the first and last stimulation day. **D**) Representative recordings and spectrograms of hippocampal LFPs in the interictal stage on the first and last stimulation day. Note that the interictal stage in AL rats was characterized by high-voltage rhythmic activity, which was significantly reduced in the IF group. Data are expressed as median ± SEM. Spectral power and frequency of LFPs were analyzed by two-way repeated measures ANOVA followed by Tukey’s post hoc test, *p < 0.05; **p < 0.01; ****p < 0.0001. Data on the relative spectral power of frequency bands were analyzed using the Kruskal-Wallis test, *p < 0.05; ***p < 0.001

**Fig. 4:**
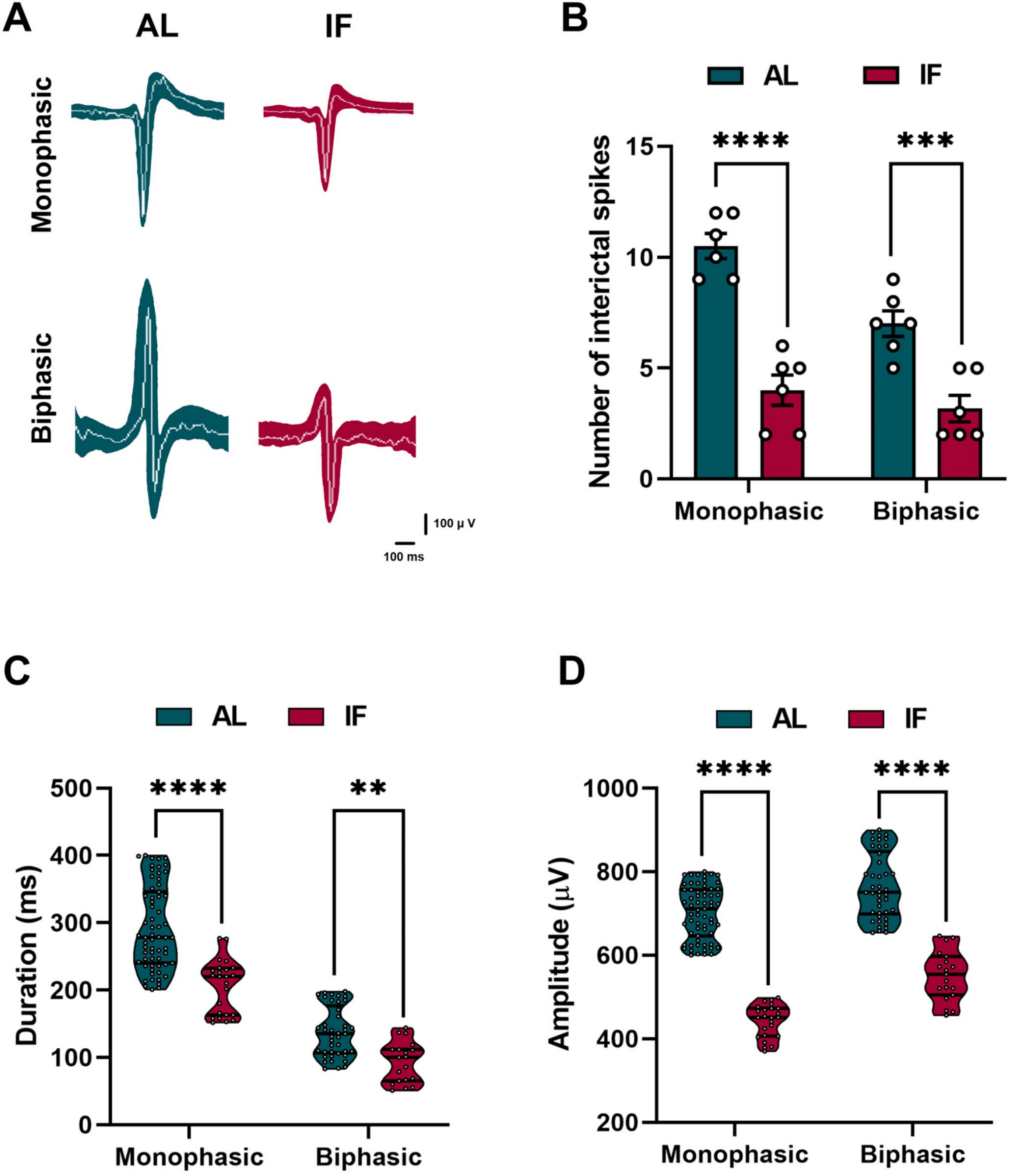
**A**) Patterns of interictal spikes recorded from the hippocampus at the end of the experimental phase. Monophasic and biphasic interictal events are shown. Monophasic and biphasic interictal events are shown within one minute. The colored area represents the magnitudes of the spikes found during analysis. **B**) Distribution of the number of interictal spikes in the hippocampus for one minute. **C**-**D**) Violin plots showing the duration and amplitude of recorded hippocampal interictal spikes. Data are expressed as median ± SEM. Two-way repeated measures ANOVA was used followed by Tukey’s post hoc test, **p < 0.01; ***p < 0.001; ****p < 0.0001

To analyze the effects of IF on the epileptogenic process, we compare LFPs obtained in stimulation 1 and 36. In both groups, LFPs recordings from the CA3 region of the hippocampus confirmed the presence of interictal epileptiform discharges, or interictal spikes, during epileptogenesis induced by electrical kindling (Fig. 2D). Interestingly, this electrical activity was not associated with any seizure behavior in either group. When we compared the LFPs recordings obtained at different stimulation days, we noticed that in the AL group, the frequency and total spectral power of the LFPs increased over time, while these effects were not observed in the IF group (Fig. 3 A and B). After examining the different frequency bands (delta, theta, alpha, and beta), we observed significant changes only in the beta band. Specifically, we found that the beta band increased in the AL group over time, but this increase was prevented by IF (Fig. 3 C). The spectrograms of the hippocampal LFPs during the interictal stage confirmed that the epileptiform discharges, which occurred after finishing the kindling protocol (stimulation 36), were constituted by high-voltage rhythmic activity (Fig. 3D). Furthermore, the recorded interictal activity was characterized by two types of spikes, monophasic and biphasic, which appeared in both the AL and IF groups (Fig. 4 A). However, the number of monophasic and biphasic interictal spikes was lower in the IF group than in the AL group (Fig. 4B). Additionally, the duration and amplitude of these spikes were found to be lower in the IF group (see Fig. 4 C and D). Overall, the IF reduces hippocampal interictal epileptiform discharges, directly modifying the evolution of the epileptogenic process.

### IF model does not produce malnutrition

**Fig. 5:**
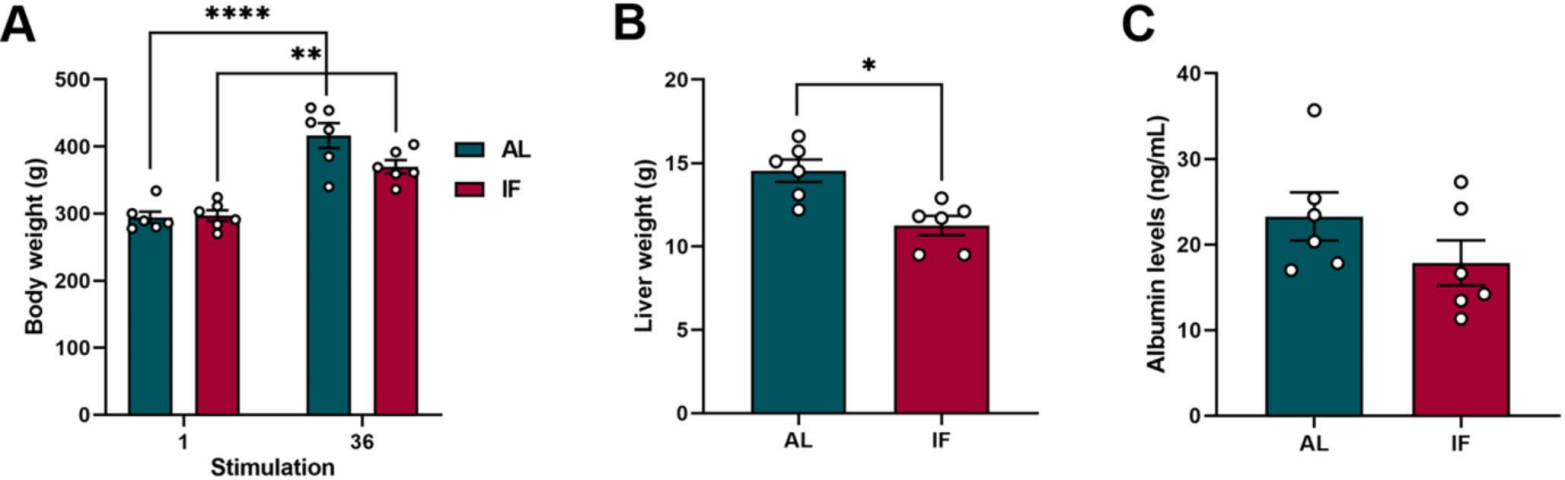
**A**) Body weight at the beginning (stimulus 1) and end (stimulus 36) of the experimental period. **B**) Liver weight at the time of animal sacrifice. **C**) Blood albumin levels at the end of the experimental period. Data are expressed as mean ± SEM. Body weight was analyzed by two-way repeated measures ANOVA followed by Tukey’s post hoc test, **p < 0.01; ****p < 0.0001. While liver weight and albumin levels were analyzed with an unpaired t test, *p < 0.05

Body weight, liver weight and plasma albumin levels were evaluated as indicators of nutritional health in the animals. Both groups showed an increase in body weight during the 36 days of stimulation, with no significant differences between them at the end of this period (Fig. 5 A). Liver weight decreased significantly with IF treatment compared to the control group (Fig. 5B). However, no significant differences were found in plasma albumin levels in both groups (Fig. 5 C). These findings suggest that, at least during the IF schedule, there is no evidence of chronic malnutrition.

### The reduction in blood glucose levels by IF is associated with antiepileptogenic effects

**Fig. 6:**
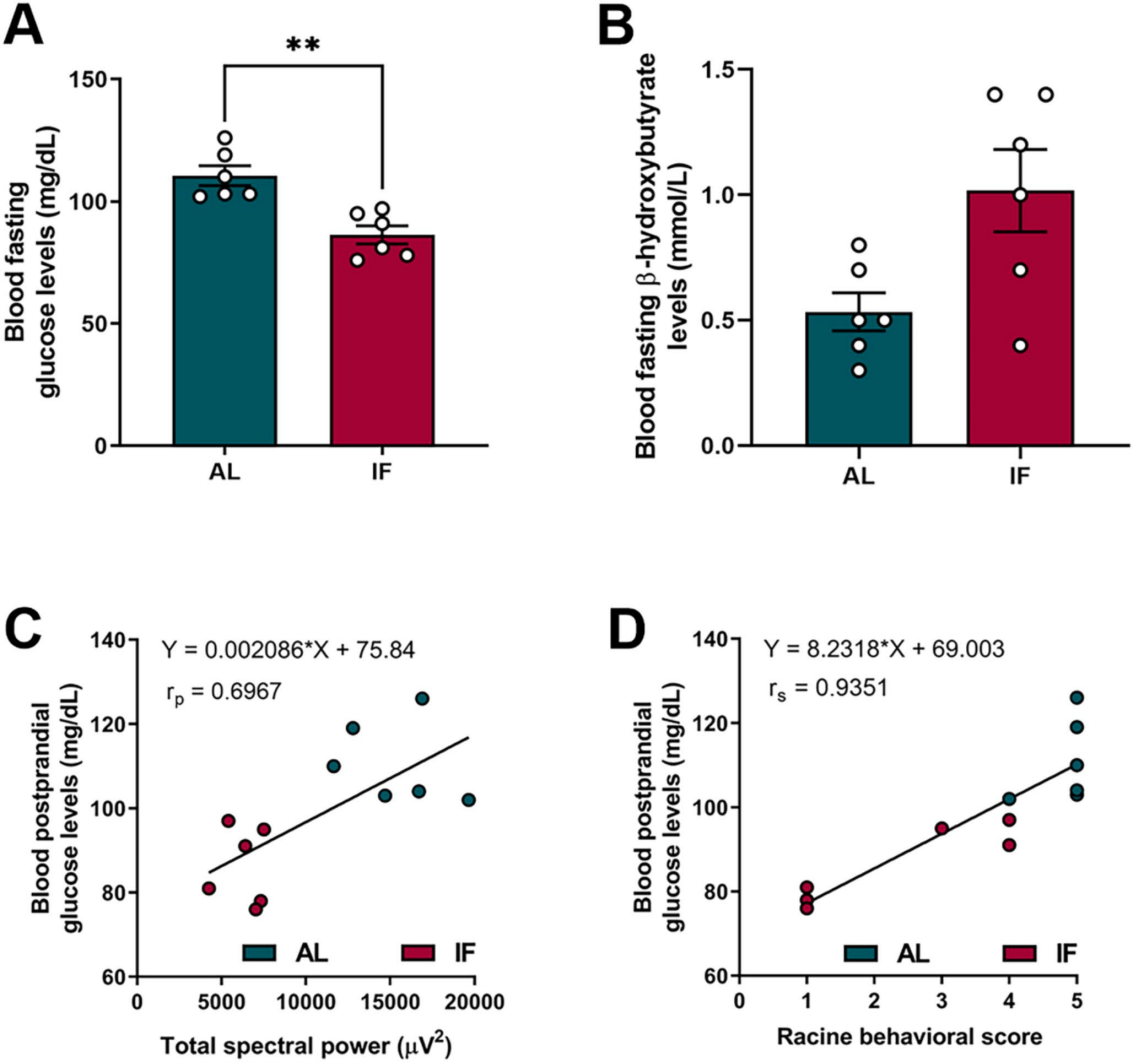
**A**) Graph of fasting blood glucose levels on the last day of stimulation. **B**) Graph of fasting blood β-hydroxybutyrate levels on the last day of stimulation. **C**) Correlation between fasting blood glucose levels and total spectral power of interictal LFPs, on the last stimulation day. **D**) Correlation between fasting blood glucose levels and behavioral score (Racine), on the last stimulation day. Data are expressed as mean ± SEM. Unpaired t-test, *p < 0.05. *n* = 12 for Pearson and Spearman​ evaluation tests, *p* < 0.05

Our results showed that IF significantly reduced glucose levels compared to the AL group (Fig. 6 A). No significant changes were observed in β-hydroxybutyrate levels between both groups, however there was a tendency to increase in the IF group (Fig. 6B). Furthermore, reduced glucose levels showed a linear correlation with the spectral power of epileptiform interictal discharges in the hippocampus (Fig. 6 C). Likewise, a linear relationship was identified between glucose levels and the Racine behavioral score achieved by the animals after stimulation (Fig. 6D). These findings suggest that the reduction in blood glucose induced by IF is significantly correlated with antiepileptogenic effects.

### IF reduces hippocampal reactive astrogliosis

**Fig. 7:**
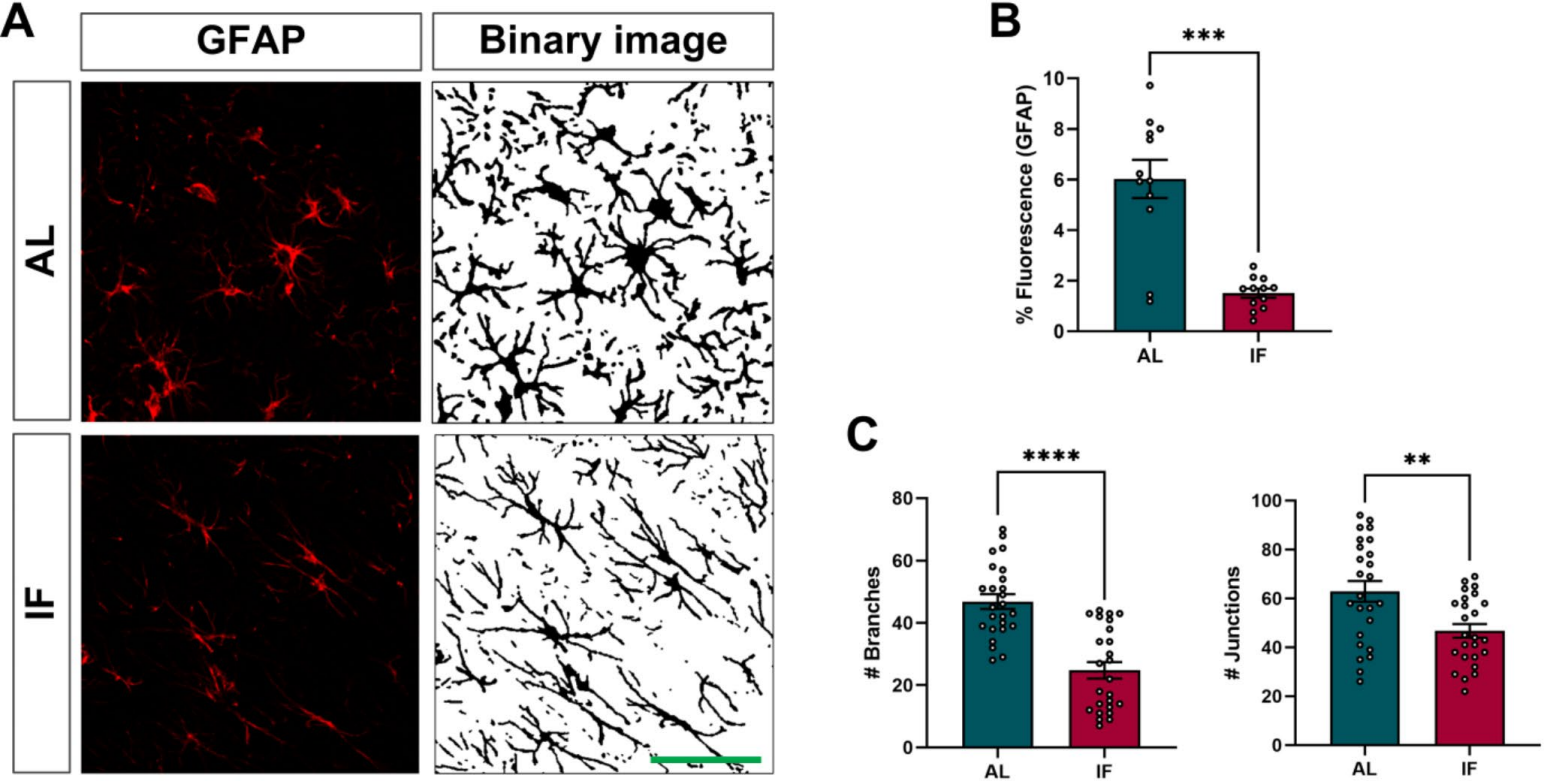
**A**) Representative photomicrographs of GFAP+ (red) labeling with the corresponding binary image of astrocytes in the CA3 region of the hippocampus contralateral to the stimulation site. Calibration bar 50 μm. **B**) Percentage of area with fluorescent signal for GFAP+. **C**) Morphological parameters of astrocytes (number of branches and junctions). Data are expressed as median ± SEM. Mann-Whitney U test. **p < 0.01; ***p < 0.001; ****p < 0.0001

Previously, we conducted a sham group on animals fed ad libitum. These animals had the electrode implanted in the hippocampus but did not receive any electrical stimulation (AL-SHAM, *n* = 6). We compared this sham with the AL group, which was stimulated for kindling. Our observations showed that electrode implantation per se increased GFAP expression due to the scar caused by the mechanical injury (Supp. Figure 1B and 1 C). We compared the AL and IF groups analyzing the astrogliosis in the contralateral hippocampus to avoid bias due to the electrode lesion. This has been previously validated because epileptiform activity spreads to the hemisphere contralateral to the stimulation during kindling (Fig. 7 A) (Chiba and Wada 1997). Our findings indicate that IF significantly reduced the GFAP expression in the hippocampal CA3 region compared to the AL group (Fig. 7B). Furthermore when comparing astrocytes from AL animals to those subjected to IF, it was observed that IF prevented increased branches and junctions. These parameters indicate astrocytes morphological complexity, suggesting that IF prevents astrogliosis (Fig. 7 C).

## Discussion

It has been shown that intermittent fasting can induce a series of metabolic and biochemical changes, such as a reduction in glucose concentration, the formation of ketone bodies, and an increase in AMP-activated protein kinase (AMPK) activity (Maalouf et al. 2009; Burkewitz et al. 2014; Longo and Mattson 2014; Yuen and Sander 2014). Our results support these findings, as we showed that IF tends to increase the β-hydroxybutyrate levels and significantly reduce blood glucose levels. Furthermore, after 36 days of IF, animals did not show significant decreases in body weight or albumin levels, although a reduction in liver weight was observed compared to animals fed ad libitum. This indicates that this IF protocol did not alter the nutritional state of the animals as other restrictive diets (Amigo and Kowaltowski 2014; Landgrave-Gómez et al. 2016; Santillan-Cigales et al. 2020). Furthermore, the induced changes in glucose homeostasis appear to be compatible with the proper functioning of brain cells. The latter is important because there is increasing evidence that metabolic-based treatments have significant anticonvulsant effects (Yuen and Sander 2014; Landgrave-Gómez et al. 2016).

In our study, we observed that the kindling model was established in rats that were fed ad libitum within 36 stimuli protocol applied in the CA3 region of the hippocampus. These animals reached score 5 of epileptic seizures according to the Racine scale. In contrast, the group that was subjected to IF showed a delay in the process of epileptogenesis. It is possible that, with prolonged stimulation, this group will also reach score 5 of epileptic seizures, as suggested by Brandt and colleagues (2004), nevertheless, it will be interesting to study this phenomenon more exhaustively.

Interictal discharges are electrographic changes observed in the electroencephalogram (EEG) in the absence of seizures. That is, they occur when patients affected by epilepsy are asymptomatic (Staley et al. 2011). However, there is still a debate as to whether these interictal spikes are precursors or causes of epilepsy (Lai et al. 2023). This is due to the existence of studies that record interictal discharges just after an induced epileptic state occurs (Bortel et al. 2010). Recent studies suggest interictal epileptiform discharges emerge in the hippocampus during epileptogenesis in animal models (Fisher et al. 2014; Kalogeropoulos and Psarropoulou 2024). Interictal epileptiform discharges are essential in the epileptogenic process because they facilitate abnormal synchronization of the hippocampus with other related structures, such as the cortex. This recruitment of more brain areas leads to the propagation of epileptiform activity (Gelinas et al. 2016; Kalogeropoulos and Psarropoulou 2024). The electrical stimulation facilitates the appearance of interictal epileptiform discharges in LFPs recordings, which is consistent with previous reports describing interictal spikes in the CA3 area (Cohen et al. 2002). This reinforces the hypothesis that hippocampal hyperexcitability is essential for establishing epileptogenesis (Staley and Dudek 2006). Furthermore, it has been proposed that different hippocampal and parahippocampal regions have a differential susceptibility to epileptogenesis (Aubert et al. 2016). We corroborated the above because the animals implanted outside the target region (CA3) did not show evident interictal epileptiform discharges in the LFPs recordings (data not shown).

Most studies using kindling as an epileptogenic model focus only on evaluating and characterizing the ictal stage. However, this approach ignores interictal LFPs, resulting in a loss of valuable information about the electrophysiological mechanisms of epileptogenesis that could help predict the onset of epileptic seizures (da Silva Lourenço et al. 2021; Abe et al. 2022). In the present work, it was found that IF prevented hippocampal hyperexcitability during the interictal stage by reducing the expression of interictal epileptiform discharges. This could be verified because once the epileptogenic process was established (stimulus 36), we observed a significant decrease in the total spectral power and frequency in the LFPs recordings. Interestingly, similar inhibition of the interictal epileptiform activity has been reported after introducing ketogenic diet treatment in children with drug-resistant epilepsy (Kessler et al. 2011). The antiepileptogenic effects of IF observed on hippocampal LFPs were accompanied by an evident reduction in the beta-band. Previous studies have reported that the beta band increases in the preictal stage of epileptic patients and that its increase may be an indicator of seizure onset (Sharma 2015). Conversely, lower beta power has been found to correlate with more extended seizure-free periods (Song et al. 2019).

Based on our results where we found a reduction in blood glucose levels with IF, in the present study, we addressed the hypothesis that the antiepileptogenic effect of IF could be related to a reduced blood glucose level. Glucose is the primary source of energy for brain cells. During seizures, hyperexcitability of neuronal networks is associated with increased glucose uptake and metabolism (Ivanov et al. 2015; Boison and Steinhäuser 2018; Rho and Boison 2022). Moreover, Zhao et al. (2022) and collaborators showed that elevated concentrations of brain lactate and glucose, among other metabolic changes, may contribute to the development and progression of seizures. In this regard, we found that IF decreases the glucose levels in our preclinical model. IF-induced disturbance of glucose metabolism could affect astrocytes’ function because these cells transport and deliver nutrients to neurons and control cerebral blood flow in a manner dependent on energy activity (Beard et al. 2022).

Furthermore, we can hypothesize that IF potentially downregulates the energy supply in the brain, leading to the disruption of processes with high energy demand, such as epileptogenesis and ictogenesis, produced by a kindling stimulation protocol.

Reducing the glucose available for uptake by astrocytes would reduce lactate synthesis, preventing its function as an energy substrate in the brain (Dienel 2012). Anticonvulsant effects have been described by reducing lactate levels and ATP production, which are essential for maintaining robust epileptiform activity. Therefore, these studies have shown that inhibiting glycolysis or lactate dehydrogenase reduces extracellular lactate levels and suppresses seizures and epileptiform activity (Shao and Stafstrom 2017; Pan et al. 2019; Janicot et al. 2020; McDonald et al. 2021; Skwarzynska et al. 2023).

Under non-pathological conditions, dietary treatments such as ketogenic diet and caloric restriction have been shown to influence astrocyte morphology in the hippocampus, remodeling their structural complexity. However, studies by Gzielo et al. (2019) and Popov et al. (2020) have shown that these treatments do not generate evident astrocyte activation or an increase in GFAP expression, suggesting that IF does not induce astrogliosis under these conditions.

The observed reduction in astrogliosis in the IF group following kindling stimulation supports the hypothesis that this phenomenon may be mediated by two distinct mechanisms, or potentially by a combination of both. The first proposed mechanism suggests that IF acts directly by reducing seizure activity by a combination of reduced blood glucose levels or the presence of ketone bodies mainly β-hydroxybutyrate thus, decreasing the activation of astrocytes. Secondly, seizure activity triggers brain inflammation through the participation of astrocytes, promoting an increase in the synthesis of GFAP and modifications in astrocytic morphology, such as the increase in the number and extension of their processes, changes in characteristics of the process of epileptogenesis (Díaz et al. 2023; Tewari et al. 2023). Although we did not find a significant increase in β-hydroxybutyrate, we can speculate that the presence of this ketone body could have an anti-inflammatory effect as observed in the literature thus preventing astrogliosis (Santillan-Cigales et al. 2020).

It would be valuable to investigate GFAP expression during the epileptogenic phase to more accurately assess the effect of IF on astrogliosis. This would allow determining whether the ability of IF to reduce GFAP immunoreactivity responds to direct modulation of epileptogenic mechanisms or metabolic changes in glial cells induced by hypoglycemia.

Furthermore, these findings give rise to a novel question: how might reduced gliosis modulate the expression of beta activity? particularly with epileptiform interictal discharges. Addressing this question could offer a deeper understanding of the mechanistic effects of IF on brain activity and its potential therapeutic relevance. To date, no studies have been reported that specifically analyze the relationship between astrogliosis and EEG beta activity. It is well-known that astrocytes play a fundamental role in neuronal excitability (Verhoog et al. 2020).In this regard, it has been described that astrogliosis can contribute to the development of spontaneous seizures (Robel et al. 2015). However, to the best of our knowledge, this is the first study to propose a potential association between astrogliosis and EEG beta activity. This finding is particularly significant given the documented alterations in beta rhythm activity in patients with temporal lobe epilepsy (Green and Wilson 1961).

Our results show that IF decreases GFAP immunoreactivity, a key marker of astrogliosis, and reduces the structural complexity of astrocytes compared to ad libitum-fed animals. These findings are consistent with previous studies, which reported that daytime-restricted feeding decreased reactive astrogliosis after pilocarpine-induced seizures (Santillán-Cigales et al. 2020).

Although the metabolic and morphological changes described here could contribute to the antiepileptogenic effect of IF, other signaling pathways may also play a relevant role. Mechanisms such as inhibition of the mammalian target of rapamycin (mTOR), activation of AMP-dependent kinase (AMPK) or modulation of the NLRP3 inflammasome (Balasse 1979; McDaniel et al. 2011; Yuen and Sander 2014; Youm et al. 2015) could be involved in the reduction of interictal epileptiform discharges and ictal activity induced by the kindling model, contributing to the neuroprotective effect of IF.

## Conclusions

The data presented here suggest that IF may be a feasible dietary treatment for reducing interictal epileptiform discharges and generalized seizures during hippocampal epileptogenesis and this antiepileptogenic effect of IF may be related to a hypoglycemic state, and inhibition of reactive astrogliosis in the hippocampus. Finally, it would be recommended to investigate the optimal level of blood glucose for ensuring the proper functioning of brain cells, while also determining the feasibility of using the IF schedule as an alternative treatment for drug-resistant epilepsy.

## Supplementary Information

Below is the link to the electronic supplementary material.

Supp. Figure 1. A) Diagram of the ventral hippocampus showing the sites where the stimulation electrode was placed. The red box indicates the area where the micrographs for GFAP + analysis were taken in the contralateral hippocampus of the experimental groups (AL and IF). Part of the path left by the recording and stimulation electrode is also shown using Nissl staining. B) Percentage of area with fluorescent signal for GFAP + of the stimulation electrode placement site in AL-SHAM and AL-Kindling rats. Data are expressed as the median ± SEM. No significant differences were found using the Mann-Whitney U test. C) Representative photomicrographs of GFAP+ (red) labeling of the stimulation electrode placement site in AL-SHAM and AL-Kindling rats. Please notice that in both groups, the GFAP mark left by the stimulation and recording electrode is identical. Calibration bar 200 µm

High Resolution Image (TIF 1.93 MB)

## Data Availability

No datasets were generated or analysed during the current study.
