# Supplementary figures and images for: Intermittent fasting reduces interictal epileptiform discharges and hippocampal reactive astrogliosis during electrical kindling epileptogenesis
Source: Metab Brain Dis. 2025 Apr 15;40(4):182. doi: 10.1007/s11011-025-01607-9 (PMC12000216; doi:10.1007/s11011-025-01607-9)

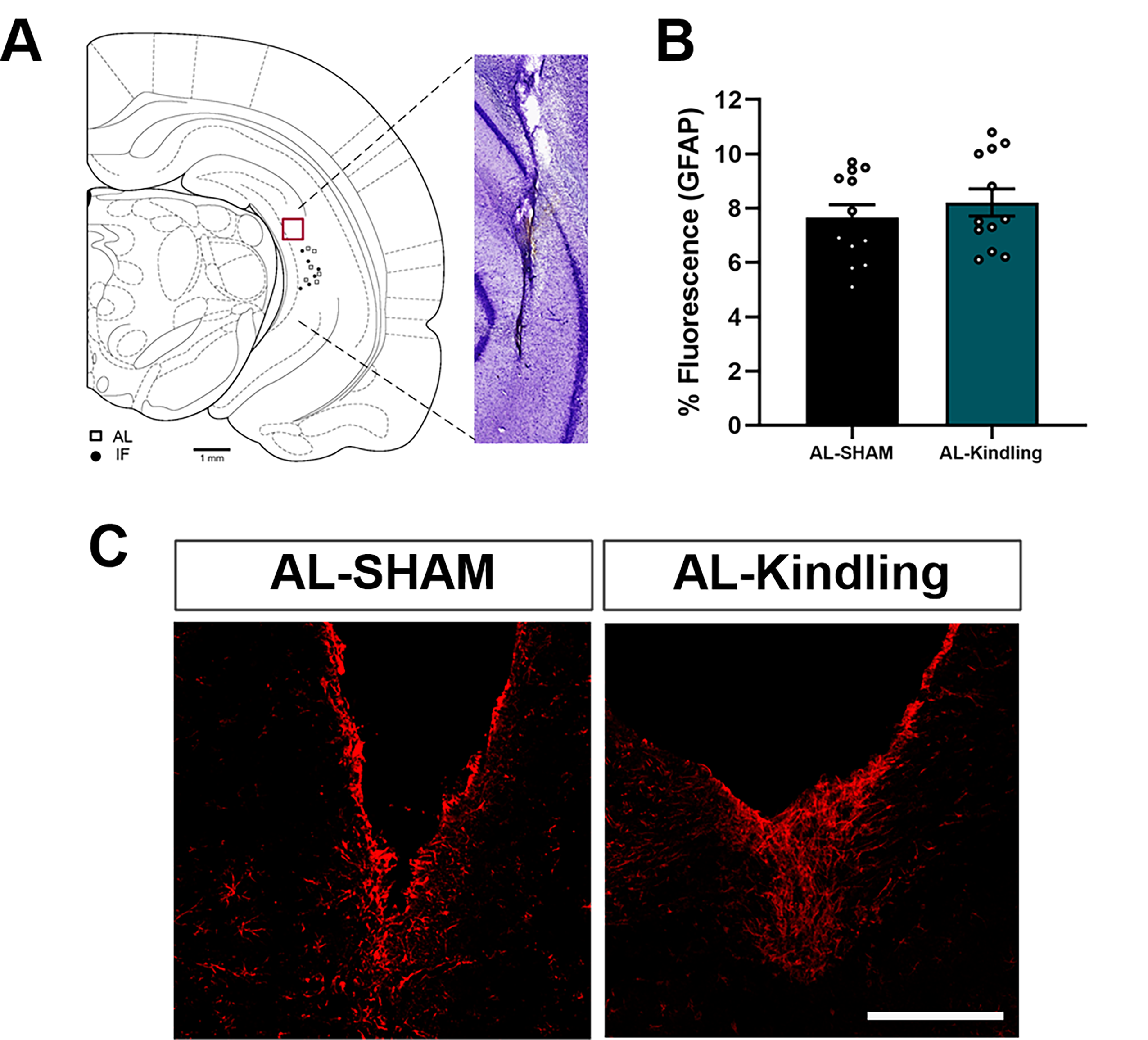

Supplement: Supplementary file 1 — Supp. Figure 1. A) Diagram of the ventral hippocampus showing the sites where the stimulation electrode was placed. The red box indicates the area where the micrographs for GFAP + analysis were taken in the contralateral hippocampus of the experimental groups (AL and IF). Part of the path left by the recording and stimulation electrode is also shown using Nissl staining. B) Percentage of area with fluorescent signal for GFAP + of the stimulation electrode placement site in AL-SHAM and AL-Kindling rats. Data are expressed as the median ± SEM. No significant differences were found using the Mann-Whitney U test. C) Representative photomicrographs of GFAP+ (red) labeling of the stimulation electrode placement site in AL-SHAM and AL-Kindling rats. Please notice that in both groups, the GFAP mark left by the stimulation and recording electrode is identical. Calibration bar 200 µm [file 11011_2025_1607_Fig8_ESM.png]

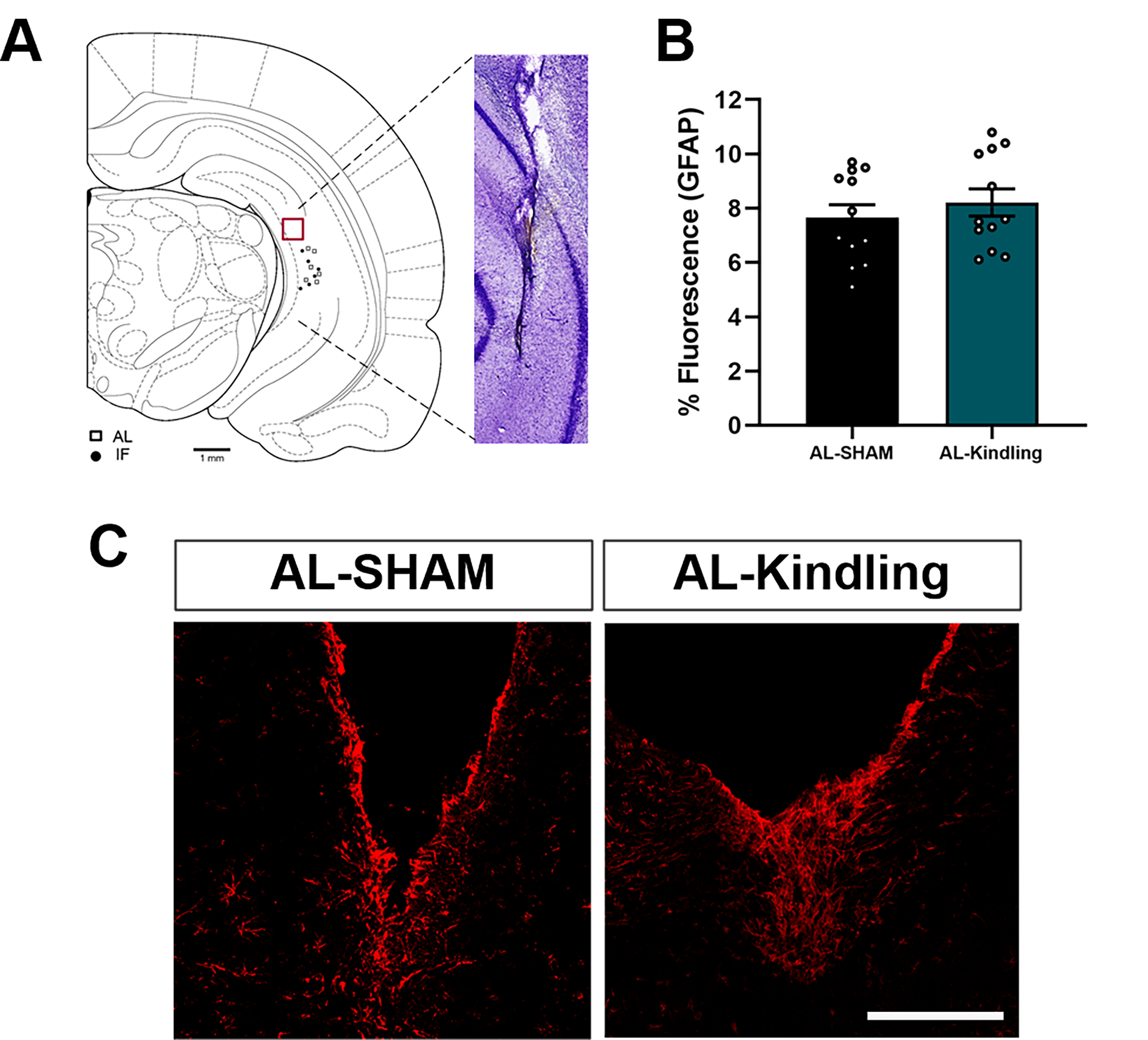

Supplement: Supplementary file 2 — High Resolution Image (TIF 1.93 MB) [file 11011_2025_1607_MOESM1_ESM.tif]
